# Therapeutic effect of fecal microbiota transplantation on rats with liver cirrhosis and its influence on gut microbiota

**DOI:** 10.22038/IJBMS.2024.74280.16142

**Published:** 2024

**Authors:** Rongrong Chen, Fangmei Wu, Guannan Zeng, Yuanchun Chen, Shiyun Lu, Huping Huang

**Affiliations:** 1 Provincial Clinical Medical College of Fujian Medical University, Fuzhou 350001, Fujian, China; 2 Department of Gastroenterology, Fujian Provincial Hospital South Branch, Fuzhou 350028, Fujian, China; 3 Department of Gastroenterology, Fujian Provincial Hospital, Fuzhou 350001, Fujian, China; # These authors contributed equally to this work

**Keywords:** 16S rRNA, Fecal microbiota- transplantation, Gut microbiota, Intestinal flora, Liver cirrhosis

## Abstract

**Objective(s):**

This study aimed to explore the therapeutic effect of fecal microbiota transplantation (FMT) on liver cirrhosis-induced rat models by studying changes in intestinal flora distribution and liver pathology.

**Materials and Methods:**

Cirrhosis was induced in adult male Sprague-Dawley rats using carbon tetrachloride; successful establishment of the cirrhosis model was verified using hematoxylin and eosin (HE) staining. Rats were divided into normal control, cirrhosis model+normal saline, and cirrhosis model+FMT groups. Fecal intestinal flora was analyzed using 16S rRNA high-throughput sequencing for each group. Alpha diversity, beta diversity, and functional prediction analyses were performed. Additionally, rat liver tissue was subjected to HE staining to compare the degree of fibrosis and liver damage between the groups.

**Results:**

FMT significantly improved the diversity, richness, and uniformity of the intestinal flora in rats with liver cirrhosis. Notably, post-FMT, the abundance of lactobacillaceae, bacilli, and bacteroidia increased, while the abundance of clostridia decreased. Moreover, hepatic fibrosis improved after FMT.

**Conclusion:**

The dysbiosis of intestinal flora in rats with liver cirrhosis improved after FMT. Thus, FMT can regulate intestinal flora, reduce liver inflammation, and improve hepatic fibrosis and cirrhosis.

## Introduction

Cirrhosis, the end stage of various chronic liver diseases, is characterized by diffuse hepatic fibrosis and replacement of normal liver architecture with nodules. It is currently the 11th leading cause of mortality, accounting for 3.5% of all deaths worldwide (1). Complications such as portal hypertension, hepatic encephalopathy, hepatocellular carcinoma, and sepsis are common causes of mortality in patients with decompensated cirrhosis (2). 

The gut microbiota is intricately intertwined with the regulation of human health, with deviations in its composition being associated with a myriad of diseases, including obesity, diabetes, chronic liver disease, inflammatory bowel disease, immune system disorders, and several types of cancer (3). Moreover, the gut-liver axis plays a significant role in the pathogenesis of cirrhosis because the liver is the first organ to encounter bacteria emanating from the gastrointestinal tract. Chronic liver diseases are characterized by significant microbial alterations, including reduced species diversity and bacterial overgrowth. Thus, it is unsurprising that patients with cirrhosis often experience enteric bacterial dysbiosis, altered gut permeability, and bacterial translocation across the gut epithelial barrier (4). 

Interventional studies using prebiotics, probiotics, and antibiotics have validated the crucial role of gut microbiota in cirrhosis development (5). Recently, fecal microbiota transplantation (FMT) has gained popularity as a treatment option for patients with chronic liver disease and cirrhosis (6). FMT involves the transfer of healthy donor microbiota to the recipient, thereby restoring dysbiotic microbial communities and facilitating the establishment of complex and resilient microbial ecosystems (7, 8). In clinical practice, FMT has shown remarkable success in the treatment of *Clostridium difficile* infections, with a cure rate of 90% (9, 10). Likewise, the first controlled study of FMT in patients with liver cirrhosis demonstrated significant improvements in cognitive function and reduced hospital stay, attributed to beneficial changes in the gut microbiota composition, including increased microbial diversity (11).

The exact mechanisms underlying the interactions between the gut microbiota and liver cirrhosis, as well as its associated complications, remain unclear. Emerging evidence suggests the involvement of various factors, such as bile acids, endotoxins, and bacterial translocation (12). For instance, declining liver function due to cirrhosis leads to decreased bile acid secretion into the intestines, weakening the inhibitory effect on potential pathogenic microorganisms and resulting in pathogenic bacteria overgrowth in the gut. These microbial changes further exacerbate intestinal inflammation and damage the intestinal mucosal barrier, inhibiting bile acid secretion in the liver, and creating a vicious cycle (13). Additionally, intestinal tight junctions are disrupted during cirrhosis development, which permits bacterial translocation and passage of bacterial products (such as lipopolysaccharides and endotoxins) into the liver. These products can be recognized by pattern recognition receptors, such as toll-like receptors, leading to the activation of nuclear factor-kappa B expression and subsequent systemic inflammatory responses (14). This cascade of events contributes to the occurrence of infections and other complications and exacerbates liver damage via activation of stellate cells (15). Furthermore, bacterial translocation, endotoxemia, and pro-inflammatory cytokines also impair the contractility of mesenteric vessels and increase portal pressure (16).

Given the increasing global prevalence of cirrhosis, it is imperative to enhance our understanding of the relationship between gut microbiota and cirrhosis development to improve prognosis and design effective therapeutic strategies. Therefore, in this study, we aimed to investigate the therapeutic effects of FMT by analyzing the stool flora and comparing the liver pathology of cirrhotic rats before and after FMT.

## Materials and Methods

***Ethical considerations***

All experimental procedures conformed to the 3R principles for experimental animals and were approved by the Animal Ethics Committee of the School of Basic Medicine, Fujian Medical University (Animal Experiment approval number: SYXK(Min)2022-0003). 

***Animal model***

Fifty male Sprague-Dawley (SD) rats of specific pathogen-free level, weighing 200-250 g, were maintained in individually ventilated cages at an ambient humidity of 50±10% and a temperature of 20±5 ^°^C at the Experimental Animal Center of the Basic Medical College. They were maintained under a 12-hr light/dark cycle, with free access to food and water and sufficient space for all activities. Based on their initial body weight, the rats were randomly divided into three groups: a normal control group (control group), a liver cirrhosis group (CCl_4_ only group), and a liver cirrhosis treatment group (CCl_4_+FMT group). Each group consisted of 10 rats. Additionally, 10 SD rats were used as FMT fecal bacteria donors, while another 10 SD rats were used as the pre-experiment group. To generate the CCl_4_-induced liver cirrhosis model, the rats underwent a preparative period of adaptive feeding and phenobarbital drinking water intervention (one week each), after which they were injected with 50% carbon tetrachloride (CCl_4_) solution (1:1 CCl_4_ solution and olive oil) twice a week to induce liver cirrhosis. The injection dose into the rats’ backs was 3 ml/kg in the first week, with subsequent doses of 2 ml/kg for 12 weeks. During this period, rats in the normal control group received subcutaneous injections of an olive oil solution in an equivalent volume.

***Experimental method***

*FMT intervention treatment*

The detailed protocol of fecal bacterial suspension preparation has been previously published (17, 18). The freshly excreted feces of 10 SD rats of the same strain and batch, raised as fecal flora donors, were collected by stimulating the anus of the rats every day and putting the feces into a 50 ml sterile centrifuge tube, after which they were immediately placed in an ice box for storage. After weighing the feces, sterile saline of five times the volume was added to the centrifuge tube and stirred fully to obtain a suspension. A wire filter was used for the initial filtration of food residues, followed by filtration with three layers of gauze to further remove residues. The suspension was collected in a 50 ml centrifuge tube, and the process was repeated three times. The filtrate was centrifuged at 500 rpm/min for 3 min at 4 ;e; the supernatant was collected, and this process was repeated three times. Subsequently, the mixture was thoroughly shaken, following the ratio of 50 ml of bacterial solution to 5 ml of glycerol (10%). It was then divided into 2 ml freezing tubes and stored in a -80 fr refrigerator.

FMT was initiated in rats from the CCl_4_+FMT group. The rats were intragastrically administered 10 ml/kg of FMT bacterial suspension every morning at 8 AM for 12 weeks; they were weighed weekly. During this period, the rats in the control group and the CCl_4_-only group were intragastrically administered the same dose of sterile saline.

*Sample collection and processing*

The freshly excreted rat feces were collected by stimulating the anus of the rats and then stored in Eppendorf tubes. The collected specimens were quickly transferred to a -80 ^°^C refrigerator for freezing. The rats were sacrificed through cervical dislocation and dissected to remove their livers. From each specimen, two pieces of liver tissue (0.5 cm×0.5 cm×0.3 cm) were excised from the same position in the right lobe and stored in 10% formalin solution for pathological examination.

Briefly, in accordance with previous literature (17, 18), the specimen processing steps were conducted as follows: stored fecal specimens were retrieved from the CB, CA, MB, MA, MTB, and MTA groups. CB and CA refer to the normal control groups, which did not receive any intervention but were assessed for comparison before and after the experimental groups received their respective interventions; MB and MA represent the liver cirrhosis groups before and after normal saline treatment, respectively; and MTB and MTA refer to the liver cirrhosis groups before and after fecal microbiota transplantation, respectively. Three fecal specimens from each group were chosen for 16S rRNA sequencing. The fecal suspension was sequentially filtered through self-made sterile stainless-steel filters with diameters of approximately 1.0, 5.0, and 25 mm. The suspension was then distributed into 15 ml centrifuge tubes and centrifuged at 2000 r/min for 5 min, followed by discarding the supernatant. The bacterial pellet was then resuspended in sterile physiological saline to the original volume, thoroughly mixed, and centrifuged again (2000 r/min, 5 min), repeating this process three times. Finally, the bacterial suspension was mixed with glycerol (10%) at a ratio of 50 ml bacterial solution to 5 ml glycerol, aliquoted into 2 ml cryotubes, and stored at -80 ^°^C in a freezer.

*Intestinal flora sequencing*

Stool genomic DNA was extracted using an EZNA Stool DNA Kit (Omega Bio-tek, Inc., USA) following the manufacturer’s instructions. The concentration and quality of the genomic DNA were determined using a NanoDrop 2000 spectrophotometer (Thermo Scientific Inc., USA). DNA samples were stored at -20 ^°^T for subsequent experiments.

The V3-4 hypervariable region of the bacterial 16S rRNA gene was amplified using the universal primer 338F (5’-ACTCCTACGGGAGGCAGCAG-3’) and 806R (5’-GGACTACNN GGGTATCTAAT-3’)(19). For each sample, an 8-digit barcode sequence was added to the 5’ end of the forward and reverse primers (provided by Allwegene Company, Beijing, China). PCR was carried out using the Mastercycler Gradient (Eppendorf, Germany) in 25 μl reaction volumes containing 12.5 μl 2×Taq PCR MasterMix (Vazyme Biotech Co., Ltd, China), 3 μl BSA (2ng/μl), 1 μl Forward Primer (5 μM), 1 μl Reverse Primer (5 μM), 2 μl template DNA, and 5.5 μl ddH_2_O. Cycling parameters were 95. for 5 min, followed by 28 cycles of 95 fo for 45 sec, 55 5 for 50 sec, and 72 a for 45 sec, with a final extension at 72 w for 10 min. The PCR products were purified using the Agencourt AMPure XP Kit (Beckman Coulter, Inc., USA). Sequencing libraries were generated using the NEB Next Ultra II DNA Library Prep Kit (New England Biolabs, Inc., Ipswich, MA, USA) according to the manufacturer’s recommendations, and the library quality was assessed using the Nanodrop 2000 spectrophotometer, Agilent 2100 Bioanalyzer (Agilent Technologies, Inc., USA), and ABI StepOnePlus Real-Time PCR System (Applied Biosystems, Inc., USA).

Deep sequencing was performed on an Illumina MiSeq/NovaSeq (Illumina, Inc., USA) platform at Beijing Allwegene Technology Co., Ltd. Following sequencing, image analysis, base calling, and error estimation were performed using the Illumina Analysis Pipeline Version 2.6 (Illumina). The sequence data associated with this project have been deposited in the NCBI for Biotechnology Information Short Read Archive database (Accession Number: SRP******).

***Liver pathological assessment***

The Scheuer score (20) was used to grade liver damage in each group. The degree of inflammation was divided into five grades: G0, no or mild inflammation around the portal area, no inflammation in the lobules; G1, inflammation around the portal area, inflammation but no necrosis in the lobules; G2, mild inflammation around the portal area; degree of fragmented necrosis, with focal necrosis or eosinophilic bodies in the lobules; G3, moderately fragmented necrosis around the portal area, with severe focal necrosis in the lobules; and G4, severely fragmented necrosis around the portal area, with bridging necrosis. The degree of fibrosis was divided into five stages: S0 stage, no fibrosis; S1 stage, portal area expansion; S2 stage, fibrosis around the portal area, lobular structure retention; S3 stage, fibrosis with lobular structure disorder without cirrhosis; and S4 stage, liver cirrhosis.

## Results

***Liver fibrosis improved in cirrhotic rats after fecal microbiota transplantation***

**Figure 1:**
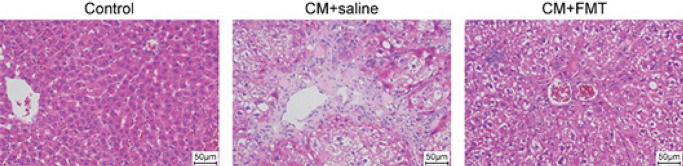
Hematoxylin and eosin (HE) staining of liver tissue of rats with control liver cirrhosis and rats with fecal microbiota transplantation (FMT)

Hematoxylin and eosin staining was used to assess the therapeutic effect of FMT on liver fibrosis in cirrhotic rats. The results demonstrated significant fibrosis of liver tissue, inflammatory cell infiltration, and collagen hyperplasia in the CCl_4_-only group (Figure 1). In contrast, collagen hyperplasia was significantly reduced, and fibrosis improved in the CCl_4_+FMT group compared to the CCl_4_-only group.

***Operational taxonomic units distribution and alpha and beta diversity analysis***

**Figure 2:**
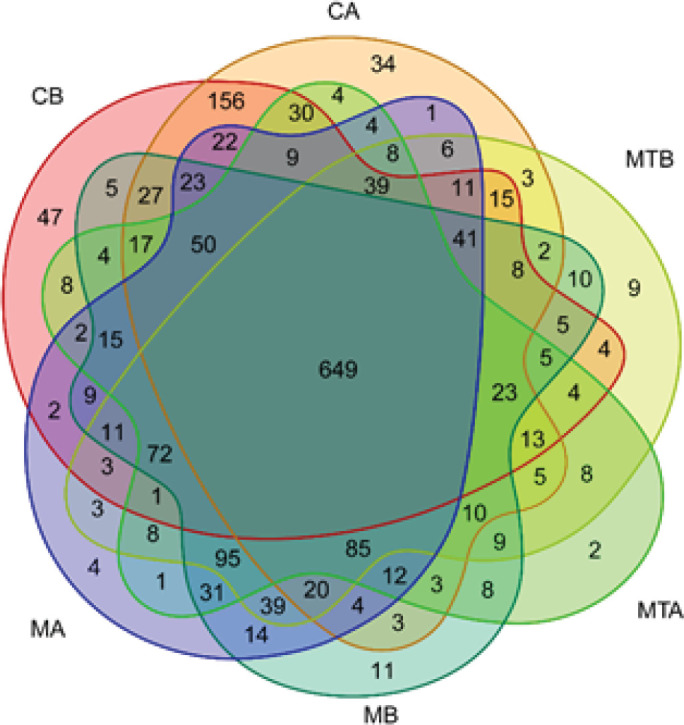
Venn diagram analysis of rats with control liver cirrhosis and rats with fecal microbiota transplantation (FMT)

**Figure 3:**
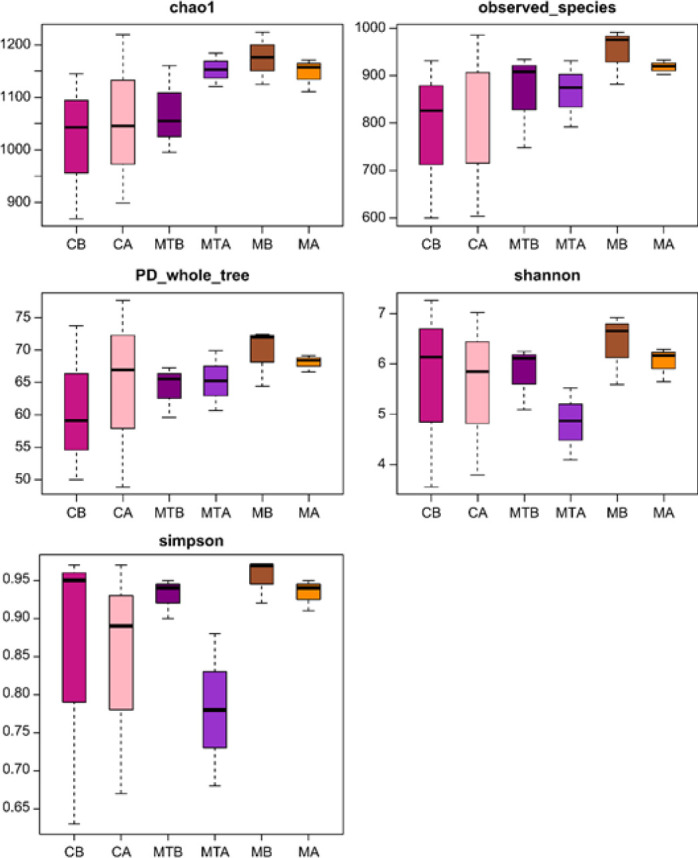
Boxplot of alpha diversity index of rats with control liver cirrhosis and rats with fecal microbiota transplantation (FMT)

**Figure 4:**
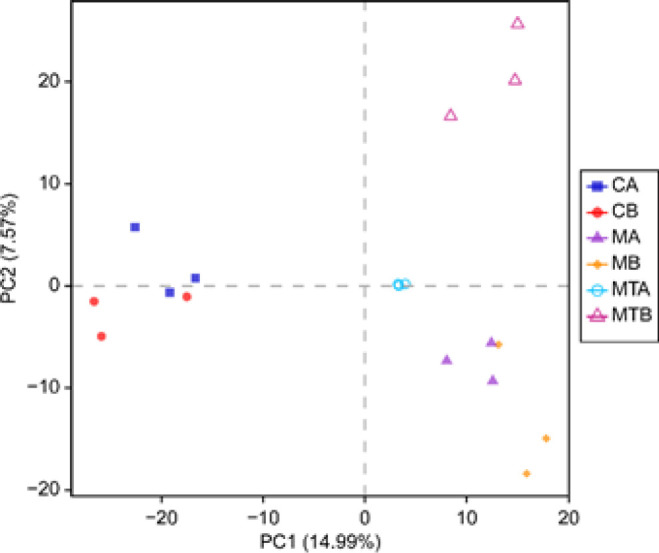
PCA analysis based on OTU level of rats with control liver cirrhosis and rats with fecal microbiota transplantation (FMT)

The CB, CA, MB, MA, MTB, and MTA groups shared 649 operational taxonomic units (OTUs)(Figure 2). Additionally, the richness and uniformity of bacterial species increased after FMT (MTA>MTB), as shown in Figure 3. Furthermore, the repeatability between the MTA groups was good, and the difference between the MTA and MTB groups was obvious (Figure 4).

***Species composition analysis and relative abundance***

**Figure 5:**
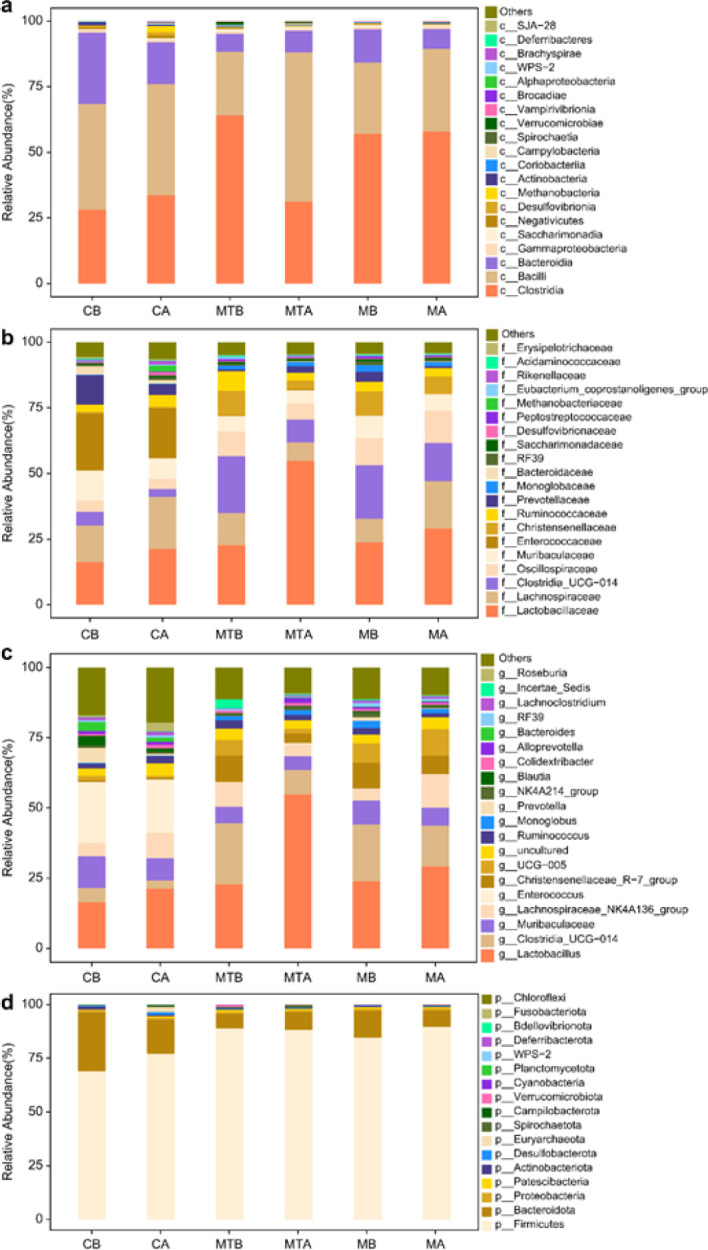
(a) Histogram of species composition analysis (Class). (b) Histogram of species composition analysis (Family). (c) Histogram of species composition analysis (Genus). (d) Histogram of composition analysis (Phylum)

A species composition analysis at the class, family, genus, and phylum levels was performed, and the data are represented as histograms (Figure 5). Compared to the control group, the number of bacilli and Bacteroidia decreased in the cirrhosis group (42.4% to 27.2%; 27.1% to 7.6%, respectively), while after FMT, there was a significant increase in both groups to reach 56.7% and 8.4%, respectively. Additionally, the abundance of Clostridia increased in the cirrhosis group (from 28.1% to 58.0%) and decreased after FMT (31.3%). Lactobacillaceae abundance increased from 22.7% to 54.7% after FMT treatment, while Clostridiaceae UCG-014 abundance increased in the cirrhosis group (5.2% to 14.7%) compared to that in the control group and decreased to 8.8% after FMT. Compared with the control group, Clostridia_UCG-014 abundance increased to 21.8% in the cirrhosis group, although after FMT intervention, it dropped to 8.8%. Finally, from the phylum level, Firmicutes accounted for more in the MTB group, reaching an abundance of 88%, while Bacteroidetes accounted for 8%.

***Operational taxonomic units and taxonomic heatmap analysis***

A heatmap can use color changes to reflect data information in a two-dimensional matrix or table and can intuitively express the size of the data value with the defined color depth. The data are often clustered according to the abundance similarity between species or samples, and the clustered data are displayed on the heatmap. High-abundance and low-abundance species can be aggregated in blocks, and the color gradient and similarity can be used to identify the similarities and differences in the community composition of multiple samples at each taxonomic level.

**Figure 6:**
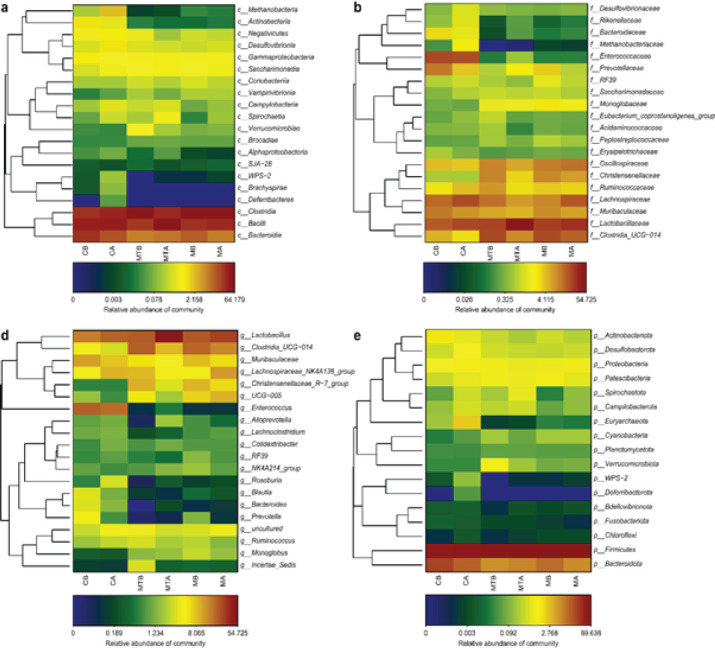
(a) On the left is the relationship cluster analysis of the species/OTUs. The abscissa is the sample name, the ordinate is OTU/species, and the color depth represents the abundance of OTU/species. Only the dominant classes are present. (b) OTU and its taxonomic level heatmap (Family). Only the dominant families are present. (d) OTU and its taxonomic level heatmap (Genus). Only the dominant genera are present. (e) OTU and its taxonomic level heatmap (Phylum). Only the dominant phylum is present

Taxonomic heat map analyses were performed at the class, family, genus, and phylum levels (Figure 6a, 6b, 6d, and 6e). At the class level, bacilli accounted for the majority of the abundance (MTB: 24.1%, MTA: 56.7%), followed by Clostridia (MTB: 64.2%, MTA: 31.3%) and Bacteroidia (MTB: 6.9%, MTA: 8.4%). At the family level, Lactobacillaceae (from 22.7% to 54.7%), Clostridiaceae UCG-014 (from 21.8% to 8.8%), Lachnospiraceae (from 12.1% to 7.1%), and Oscillospiraceae (from 9.4% to 6.1%) were the major families. Notably, the abundance of Muribaculaceae fell from 5.9% to 4.9%. At the genus level, the abundance of Lactobacillus increased from 22.7% to 54.7% after FMT. Compared to before FMT treatment, Clostridia UCG-014 and Lachnospiraceae_NK4A136_group decreased from 21.8% to 8.8% and 8.8% to 4.3%, respectively, after FMT treatment. Finally, at the phylum level, bacteria that accounted for the majority of the population were Firmicutes (88.1%), followed by Bacteroidetes (8.3%) and Patescibacteria (0.8%).

***Linear discriminant analysis effect size***

**Figure 7:**
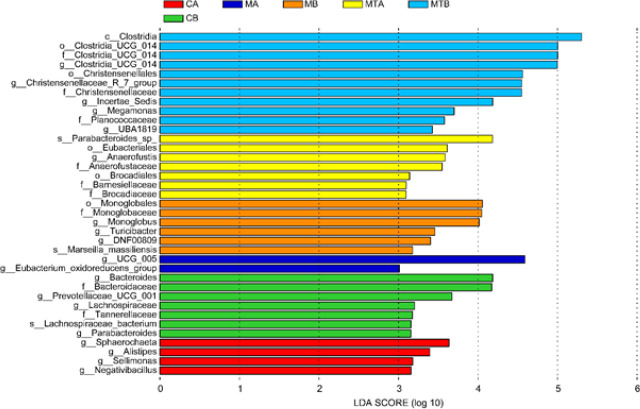
Histogram of LEfSe analysis based on classification information of rats with control liver cirrhosis and rats with fecal microbiota transplantation (FMT)

A comparison of abundance between multiple groups was performed, and a subgroup comparison analysis was carried out within a group comparison to identify species with significant differences in abundance between groups. As shown in Figure 7, in the MTB group, there were significant differences in abundance among species such as Clostridia, Clostridia UCG-014, Christensenellales, and Incertae-sedis. 

***PICRUSt and functional prediction***

**Figure 8:**
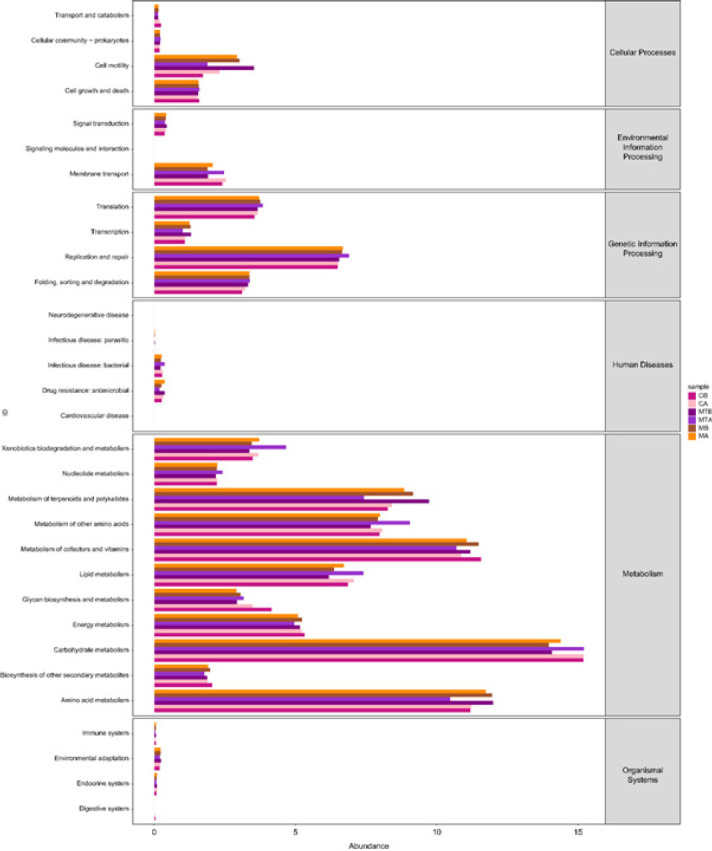
Functional predictions

As shown in Figure 8, the normal control group rats exhibited higher levels of carbohydrate metabolism, which was comparatively reduced in the liver cirrhosis group. However, after FMT, carbohydrate metabolism improved, whereas amino acid metabolism decreased.

The Venn diagram of OTU distribution can be used to enumerate the common and unique OTUs among multiple samples. It can more intuitively express the OTU number groups in environmental samples and the overlap between samples or groups. Different colors represent different samples. The area where circles of different colors overlap is the intersection. Overlapping circles of several colors have the same OTUs, while the non-overlapping part represents the unique OTUs

OTU: operational taxonomic units

Chao1 is a bacterial species richness index used to estimate the number of OTUs in a community. Observed species: As the sequencing depth increased, the number of OTUs was observed. Pedigree diversity, as represented by the PD whole tree, is an index that considers species abundance and evolutionary distance. The larger the value, the higher the community diversity. The Shannon index was used to estimate one of the microbial diversity indices in the sample. The larger the Shannon value, the higher the diversity of the community. The Simpson index considers both richness and uniformity, and the D-value reflects the probability that two individuals are randomly selected from the same sample and belong to the same class. The D value is the total number of individuals found (Ni) for a particular species divided by the total number of individuals found (N). The D values range from 0 to 1, with 0 indicating infinite diversity and 1 indicating no diversity. Therefore, the larger the 1-D value, the higher the diversity of the community. The abscissa is the sample/group name, and the ordinate is the Alpha index

Points of different colors or shapes represent different sample grouping situations, and the scales of the horizontal and vertical axes are relative distances, which have no practical significance

OTU: operational taxonomic units

The figure shows species with differences in LDA scores greater than the set value, that is, biomarkers with statistical differences. Species with significantly different abundances in different groups are displayed, and the length of the histogram represents the impact size of the significantly different species

## Discussion

This study demonstrated that FMT enhanced the diversity, richness, and uniformity of the intestinal flora in rats with liver cirrhosis. Furthermore, FMT was also found to regulate the structure and function of the intestinal flora (21), thereby alleviating and delaying the progression of liver cirrhosis while modulating key metabolic pathways. These findings deepen our understanding of the relationship between cirrhosis and gut flora imbalance while offering new perspectives for the development of novel treatments for cirrhosis and its complications.

16S rRNA gene sequencing revealed significant alterations in the intestinal flora of rats with liver cirrhosis compared with that of normal rats. Specifically, the diversity, richness, and uniformity of rare bacterial species were decreased in rats with liver cirrhosis, along with a reduction in the abundance of beneficial bacteria (such as Lactobacillus, Bacilli, and Bacteroidia) and an increase in harmful bacteria (such as Clostridia), consistent with the findings of Xiaoxia *et al.* (22). These results indicate that cirrhosis may lead to an imbalance in the gut microbiota, thereby exacerbating the development of cirrhosis and emergence of complications (23). 

In this study, FMT exhibited no obvious adverse reactions or complications, indicating a favorable safety profile under the experimental conditions. Additionally, FMT significantly augmented the diversity and richness of rare species in the gut microbiota while modulating their role in crucial metabolic pathways, such as amino acid metabolism and host-microbe symbiosis. Therefore, FMT shows potential as a promising and efficacious innovative therapy for liver cirrhosis and its related complications.

Currently, FMT is widely used for the treatment of various diseases, including refractory *C. difficile* infection, inflammatory bowel disease, and metabolic syndrome (24, 25). Numerous studies have highlighted the significant role of intestinal flora in the development and progression of liver cirrhosis, with the gut-liver axis acting as a critical interaction pathway. Given its potential efficacy in treating complications, FMT may become a novel therapeutic approach for patients with liver cirrhosis. 

This study was not without limitations. Firstly, it used a stool sample collected at a single setting, highlighting the need for further investigations to assess the effects of different fecal donors and various FMT strategies (such as pretreatment, mixing, and administration) on cirrhosis development. Secondly, only the short-term effects were evaluated. Thus, subsequent preclinical and clinical studies should prioritize exploring the long-term effects of FMT concerning therapeutic efficacy and safety in liver cirrhosis treatment. Finally, the occurrence and progression of liver cirrhosis is a multifaceted process influenced not only by intestinal flora imbalance but also by factors such as immune function, gene expression, and metabolic levels, which were not investigated in this study. Future research should focus on elucidating the mechanism of FMT in treating liver cirrhosis by investigating relevant signaling pathways, as well as determining the optimal timing, dosage, and frequency of FMT, and selecting the most suitable fecal donor and stool sample.

Animal studies have shown that FMT delays the progression of liver fibrosis in cirrhotic mice. This is achieved by modulating the intestinal flora, reshaping the immune microenvironment in the liver, and regulating the activation of hepatic stellate cells (26). Currently, there are various other treatment options for cirrhosis, including liver transplantation, interventional therapy, and drug therapy (27). Hence, further clinical investigations are warranted to confirm the efficacy and positioning of FMT in the treatment of cirrhosis.

## Conclusion

The dysbiosis of intestinal flora in rats with liver cirrhosis improved after FMT. Thus, FMT can regulate intestinal flora, reduce liver inflammation, and improve hepatic fibrosis and cirrhosis.

In summary, this study established a preliminary experimental foundation for utilizing FMT as a potential therapeutic intervention for liver cirrhosis and its associated complications. Nonetheless, several unresolved issues warrant further investigation. Future research should aim to strengthen the integration between basic science and clinical research to delve deeper into the mechanisms underlying the interplay between intestinal flora and cirrhosis. Additionally, studies should explore the potential applications and optimization strategies of FMT for liver cirrhosis treatment.
